# Inhibition of alternative oxidase disrupts the development and oviposition of *Biomphalaria glabrata* snails

**DOI:** 10.1186/s13071-022-05642-8

**Published:** 2023-02-17

**Authors:** Sha Xu, Yang-Wen-Qing Zhang, Mohamed R. Habib, Shi-Zhu Li, Yi Yuan, Wei Hao Ke, Ni Jiang, Huifen Dong, Qin-Ping Zhao

**Affiliations:** 1 Department of Parasitology, School of Basic Medical Sciences, Wuhan University, Wuhan, Hubei Province China; 2 Medical Malacology Laboratory, Theodor Bilharz Research Institute, Giza, Egypt; 3 National Institute of Parasitic Diseases, Chinese Center for Disease Control and Prevention, National Center for Tropical Diseases Research, WHO Collaborating Center for Tropical Diseases, Key Laboratory of Parasite and Vector Biology, Ministry of Health, Shanghai, China; 4 Hubei Center for Disease Control and Prevention, Wuhan, Hubei Province China

**Keywords:** *Biomphalaria glabrata*, Alternative oxidase, Development, Reproductive system, Oviposition, RNA interference

## Abstract

**Background:**

*Biomphalaria glabrata* is one of the main intermediate hosts of *Schistosoma mansoni*, the most widespread species of *Schistosoma*. Our previous studies proved that alternative oxidase (AOX), the terminal oxidase in the mitochondrial respiratory chain, widely exists in several species of intermediate host snails of *Schistosoma*. Meanwhile, inhibition of AOX activity in *Oncomelania hupensis* snails could dramatically enhance the molluscicidal effect of niclosamide. As a hermaphroditic aquatic mollusc, the high fecundity and population density of *B. glabrata* increase the difficulty of snail control, which is one of the critical strategies for schistosomiasis elimination. The present study aimed to investigate the possible role of AOX in the development and fecundity of *B. glabrata* snail, which could be manipulated more manageable than other species of intermediate host snails of *Schistosoma*.

**Methods:**

The dynamic expression of the *AOX* gene was investigated in different developmental stages and tissues of *B. glabrata*, with morphological change and oviposition behaviour observed from juvenile to adult snails. Furtherly, dsRNA-mediated knockdown of *BgAOX* mRNA and the AOX protein activity inhibiting was performed to investigate the effect of AOX on the development and oviposition of snails.

**Results:**

The *BgAOX* gene expression profile is highly related to the development from late juveniles to adults, especially to the reproductive system of snails, with a positive correlation of 0.975 between egg production and *BgAOX* relative expression in ovotestis of snails*.* The inhibition of *BgAOX* at the transcriptional level and AOX activity could efficiently inhibit snail growth. However, the interference at the BgAOX protein activity level led to more severe tissue damage and more significant inhibition of oviposition than at the transcriptional level. This inhibition of growth and oviposition decreased gradually with the increase in the snail size.

**Conclusions:**

The inhibition of AOX could efficiently disrupt the development and oviposition of *B. glabrata* snails, and the intervention targeting AOX at the juvenile stage is more effective for snails. This investigation explored the role of AOX in the growth and development of snails. It would benefit snail control in the future by providing a potential target while using molluscicides more efficiently.

**Graphical abstract:**

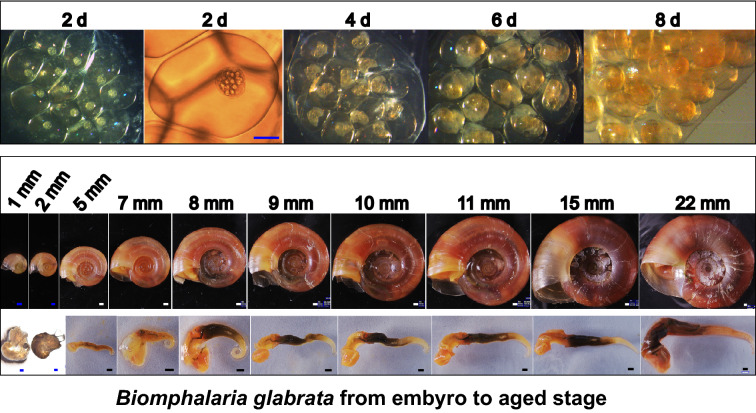

**Supplementary Information:**

The online version contains supplementary material available at 10.1186/s13071-022-05642-8.

## Background

Schistosomiasis is one of the most neglected tropical diseases, affecting almost 240 million people, with more than 700 million living in endemic areas [1, 2]. Human and other mammalian hosts are infected through skin contact with fresh water containing *Schistosoma* cercariae released from intermediate host snails [3]. Substantial progress has been made during the past decades to prevent and control schistosomiasis globally. Progress is mainly due to large-scale periodic treatment of affected populations with praziquantel, the only medicine the World Health Organization (WHO) recommended against all forms of schistosomiasis [4]. However, mass drug administration (MDA) alone may be insufficient to eliminate schistosomiasis [5]. Many approaches focused on snail control in *Schistosoma* endemic areas. Well-informed snail control could be an effective schistosomiasis control measure and serve as an essential complement to MDA in augmenting program impact [5]. Recently, WHO has been reinforcing snail control as part of its strategic approach for eliminating schistosomiasis in response to World Health Assembly resolution WHA70.16 [6].

Molluscicides (mainly niclosamide-based products) are widely used in schistosomiasis control programs. For example, schistosomiasis control programs in China largely relied on snail control for over 30 years to effectively overcome the disease. In other parts of the world, like Africa, where the MDA was the main and often the only intervention to control schistosomiasis, snail control programs gradually discontinued and even faded away. Considering China’s success story in eliminating schistosomiasis [7, 8], and at least 90% of those requiring treatment for schistosomiasis live in Africa, the China-Africa collaboration on schistosomiasis elimination was initiated [9]. After reviewing the similarities and differences in schistosomiasis control programs between China and the African continent, the effective control of intermediate host snails and precision mapping of snail distribution was suggested to be prioritised for successful schistosomiasis elimination in Africa [9]. Snail control was further indicated in global guidelines and national schistosomiasis control strategies for optimal disease control, especially in settings with high prevalence, “hot spots” of transmission, and noncompliance to MDA [10]. Meanwhile, niclosamide, the only molluscicide recommended by the WHO, has a considerable effect on snail control but is toxic to fish and other aquatic animals. It is necessary to develop new cost-effective snail control approaches that are low risk to animals and the environment*,* with the snail’s biology being much clarified.

In our previous works in enzymatic histochemistry and the metabolic and transcriptomic reaction of *Oncomelania hupensis* snail (the intermediate host of *Schistosoma japonicum*) treated with niclosamide, the decreased mitochondrial oxidative phosphorylation, the severely disturbed energy metabolism, the accumulated toxic metabolites, and damaged tissue structure were identified as the leading causes of *O. hupensis* death [11, 12]. It was noted that niclosamide induced the inhibition of aerobic respiration, the classical cytochrome c (Cyt C) pathway of mitochondrial respiratory, but the terminal oxidase of respiratory bypass in the mitochondrion, alternative oxidase (AOX), was significantly up-regulated [11–13]. Furtherly, inhibition of AOX protein activity could dramatically enhance the molluscicidal effect of niclosamide, with 56.31–76.12% higher snail mortality at 24 h post-treatment [14]. Therefore, AOX was implied to be a critical factor in maintaining metabolic homeostasis and survival of *O. hupensis* under stress [14]. The histological observation indicated that *O. hupensis AOX* (*OhAOX*) mRNA and OhAOX protein also existed in the ovarian or testicular stroma of *O. hupensis* [14]. AOX can alleviate developmental abnormalities in *Drosophila* by GeneSwitch and is involved in cell apoptosis, differentiation, and development [15]. These results led us to deduce that AOX is vital to mollusc survival under stress and may relate to snails’ growth and reproductive development under normal conditions.

AOX is widely distributed in higher plants, algae, and fungi. It also exists in some protists but not vertebrates [16, 17]. It plays roles in heat generation [16, 18], photosynthesis optimisation [19], resistance to stress [20–22], and maintenance of cellular energy homeostasis [22]. Recently, AOX was reported in several species of metazoans [17, 23], including molluscs [14, 24], but with limited information on function. Coexpression of AOX from *Ciona intestinalis* could produce a substantial shift in the developmental outcome of *Drosophila* and significantly alleviate abnormality of the cleft thorax in *Drosophila* [25]. AOX expression in *Drosophila* showed a deleterious effect on male reproductive function [26]. In addition, once a *C. intestinalis* AOX transgene was expressed in the respiratory chain complex III deficiency mouse, AOX bypass could alleviate the pathological manifestations and expand the lifespan of mice by preventing lethal mitochondrial cardiomyopathy [27]. When AOX activity was inhibited in *Trypanosoma brucei* [28], the growth and development of the protozoa were significantly inhibited, even to death [28]. These results indicate that AOX plays a role in the growth and development of certain metazoans, although the mechanism is uncertain.

AOX in *O. hupensis* was identified and confirmed to play vital roles in energy metabolism and snail survival under stress [14]. Besides, AOXs in three species of *Biomphalaria* were also identified, but the function is not clarified. *Biomphalaria* is the intermediate host snail of *Schistosoma mansoni*, the most widespread species of human *Schistosoma* distributed in 55 countries and regions [29]. *Biomphalaria* is a hermaphrodite planorbid snail capable of self- or cross-fertilisation, withstanding desiccation for months in the mud, and recolonising rapidly after the rain [30, 31], which maintains a high population density of *Biomphalaria* in the field and increases the difficulty of snail control. It is valuable to understand the biology of *Biomphalaria* snails to find a more specific way to keep the snail population at a lower density. In this study, the dynamic expression of *B. glabrata AOX* (*BgAOX*) was investigated in different developmental stages and tissues of snails, and the role of AOX in the growth and reproductive development of *B. glabrata* was specified. Furthermore, the function of AOX was clarified through RNA interference (RNAi) and AOX activity inhibitor. These suggested a specific target as a potential enhancer for developing a biocontrol strategy.

## Methods

### Snails feeding

Several *Biomphalaria glabrata* snails were kindly provided by Dr. Yousheng Liang from Jiangsu Provincial Institute of Schistosomiasis Control and Prevention. The Puerto Rico (PR) strain of *B. glabrata* has been kept and bred in Dr. Liang’s laboratory for 10 years. Snails were continued to produce for two generations in our laboratory before snails collection in this study.

Snails ranging from 10.0 mm to 15.0 mm in shell diameter were randomly selected, placed in a clean container with 1 L aerated dechlorinated tapwater per 15 snails, and reared at 26–28 °C with a 12 h light and 12 h dark photocycle and fed with fresh green-leaf lettuce. The container was cleaned regularly to remove food residue. Small clean cling films were set on the water surface to let snails deposit eggs. The egg masses were collected daily and transferred to a clean container with dechlorinated tap water. The newly hatched snails were kept in 200 ml water in a shallow 500 ml container per 100 snails and fed with dried lettuce and fish food. The juvenile and adult snails were continually transferred to a new container with 1–1.5 L of dechlorinated tap water, fed, and maintained under the same conditions as the above procedure.

### Observation and collection of snails at different developmental times

The snail age was calculated from the day the egg mass was deposited. Enough snails were collected at 1 month (M), 2 M, 3 M, and 4 M in batches from the same aquarium. Living snails were washed in sterilised tap water, and shells were cleaned with 70% ethanol. The snail was arranged with the aperture opening to the lower left, with the basal margin of the aperture paralleling the scale of the vernier calliper. The longest transverse distance was measured as the snail size in diameter, with the right angle to the columella. After the appearance of the snail, including shape, colour, and size, was observed with the 3D digital video microscope HRX-01 (Hirox, Tokyo, Japan), the snail shell was then gently peeled off, and the whole soft body was washed in 0.3% NaCl and observed under the optical microscope.

Snails of the same age from the same aquarium differ in shell diameter during their development. Then, snails from 1 to 6 M were furtherly classified into more groups based on size, including 1 M (< 1.0 mm), 1 M (1.0–2.0 mm), 1 M (2.0–3.0 mm), 1 M (3.0–4.0 mm), 2 M (2.0–3.0 mm), 2 M (3.0–4.0 mm), 2 M (4.0–5.0 mm), 2 M (5.5–7.0 mm), 3 M (3.0–4.0 mm), 3 M (4.0–5.0 mm), 3 M (5.5–7.0 mm), 3 M (7.5–8.5 mm), 3 M (9.0–10.0 mm), 3 M (10.0–11.0 mm), 3 M (12.0–13.0 mm), 3 M (14.0–15.0 mm), 4 M (16.0–17.0 mm), 5 M (18.0–20.0 mm), and 6 M (22.0–24.0 mm). Snails were washed in sterilised water, and shells were gently peeled off. The whole soft body of each snail was carefully washed three times with ice-cold DEPC-treated water containing 0.3% NaCl. Some whole soft bodies and different tissues of the same snail from each group, including the head-foot region, liver, and ovotestis, were individually dissected and immediately stored in liquid nitrogen for further RNA extraction. Some soft bodies were treated with 4% paraformaldehyde individually for histological observation. In addition, juvenile snails less than 1.0 mm (< 1 M) in diameter were gently washed in sterilised water and cleaned with ice-cold DEPC-0.3% NaCl solution, then collected as one sample per 8–15 snails and immediately frozen in liquid nitrogen.

Snails of 6.0 mm, 8.0 mm, 9.0 mm, 11.0 mm, 13.0 mm, 15.0 mm, 20.0 mm, and 24.0 mm in diameter were randomly selected and grouped. The snail was fed individually, and the egg production was counted daily for each snail for 14 days. Eight to ten snails of the same size per group were investigated. The egg production difference among groups was statistically estimated using one-way ANOVA analysis after the normality test and post hoc comparison in SPSS 21.0 software.

### RNA extraction and *BgAOX* mRNA quantification with real-time PCR

Total RNA from each sample was extracted using TRIzol Reagent (Invitrogen, California, USA) according to the manufacturer’s instructions. The integrity and size distribution of RNA was evaluated using agarose gel electrophoresis, and the RNA concentration was determined using an MD 2000 spectrophotometer (BioFuture, UK). Then, single-stranded cDNAs for each sample were synthesised from the corresponding extracted RNA using a PrimeScript™ RT Reagent Kit with gDNA Eraser (Takara, Tokyo, Japan).

The *BgAOX* gene dynamic expression profiles were evaluated in the whole snail at different ages (from less than 1 M to 6 M) and sizes (from less than 1.0–24.0 mm). Before quantitative real-time PCR (qPCR), semi-quantitative PCR was performed to confirm the presence of *BgAOX* in the whole soft body and the specificity of primers in amplification. Specific primers for partial *BgAOX* (247 bp) were synthesised as follows, AOX-F: 5′-GGATCAACCGCATTTGTTTCC-3′, AOX-R: 5′-CTGATGCCAAACCAGGTGAC-3′, based on the verified *B. glabrata AOX* complete cDNA sequence (MZ436167) [14]. Parallelly, the partial sequence of the *β-actin* cDNA gene (100 bp) in *B. glabrata* (AF435735) [32] was selected as an internal control for normalisation through preliminary tests from several reference genes, with primers as β-actin-F: 5′-CACCTCTAAACCCTAAAGCCAAC-3′ and β-actin-R: 5′-TGAGAGCACAGCTTGGATGG-3′. qPCR was performed in triplicate in a 10 μl mixture for each reaction. Each reaction was repeated five times using TB Green Premix Ex Taq II (Tli RNaseH Plus) (Takara, Tokyo, Japan) and a Bio-Rad CFX96 thermal cycler with the following protocol: 95 °C for 20 s, followed by 40 cycles of 95 °C for 5 s, 62 °C for 20 s, and 72 °C for 20 s, and melting curve analysis performed from 75 °C to 95 °C increasing by 0.5 °C at each step with a hold of 60 s. The expression profiles of *BgAOX* in snails of different ages and sizes were normalised to the expression of *β-actin* in corresponding samples using the comparative delta-delta-Ct method [33] after the amplification efficiency was adjusted to 1.89–2.18. Furthermore, the *BgAOX* expression profiles in different tissues, including the head-foot region, liver, and ovotestis, were evaluated in snails of 6.0 mm, 8.0 mm, 9.0 mm, 11.0 mm, 13.0 mm, 15.0 mm, 20.0 mm, and 24.0 mm, with the same reaction procedure and system used for the whole snail. Each reaction was performed in triplicate and repeated three to six times. All of the results in qPCR were statistically analysed among groups using one-way ANOVA analysis after the normality test and post hoc comparison in SPSS 21.0 software.

### The histological observation of snails in different developmental stages

Based on the *BgAOX* expression profiles in different stages of snail in qPCR, snails of specific sizes were selected, including 5.5–7.0 mm, 7.5–8.5 mm, 9.0–10.0 mm, 10.0–11.0 mm, and 12.0–13.0 mm, to observe the histological development of snails with conventional hematoxylin–eosin (HE) method as follows. The whole soft body of the snail was laid on the glass slide individually, 4% paraformaldehyde was added, and each snail was cut into three parts under an optical microscope, covering the head-foot, liver, and ovotestis, respectively. Tissues were fixed in 4% paraformaldehyde at 4 °C over 24 h, embedded in paraffin, and sectioned into 4 μm-thick specimens. Conventional HE staining on slides was performed continually by deparaffinisation with xylene, hydration in graded ethanol, nuclear staining in hematoxylin, tap water cleaning, differentiation and blueing, counterstain in eosin, dehydration in graded ethanol, clearing with xylene, drying and sealing with neutral balsam.

### The correlation analysis between the egg production and *BgAOX* expression profile in ovotestis of snails

According to the summarised data of two variables, the expression of *BgAOX* in the ovotestis of snails and the average egg production daily per snail in different sizes, Pearson correlation analysis was performed in SPSS 21.0 with the correlation coefficient (*r*) setting as the following criterion: − 1 ≤ *r* < 0 indicates a negative correlation and 0 < *r* ≤ 1 indicates a positive correlation between variables, respectively, and *r* = 0 means no linear correlation, but a nonlinear relationship may exist. The |*r*| > 0.8, 0.5 <|*r*| ≤ 0.8, 0.3 < |*r*| ≤ 0.5, and |*r*| ≤ 0.3 were further set as high, moderate, low, and no correlation, respectively.

### The effect of *BgAOX* RNAi and AOX activity inhibitor on the growth and reproductive development of snails

Double-stranded (ds) RNA-mediated RNAi of *BgAOX* was used to silence *BgAOX* gene expression. Parallelly, the AOX inhibitor, salicylhydroxamic acid (SHAM), was used to inhibit AOX activity in snails. Then, the effect on snails’ growth and reproductive development was investigated.

### dsRNA preparation

After blast analysis and high similarity screening (> 19 bp) with *B. glabrata* genome sequence, sequences of 380 bp (spanned position 141–530) and 553 bp (spanned position 961–1530) on *BgAOX* cDNA were selected as the target fragments of RNAi and named as BG380 and BG553, respectively. The specific primers were designed by adding protective base ATTT and *Not*I restriction site to the forward primers BG380-F: 5′- ATTTGCGGCCGCTTTATGATCCGAATTTTATAGGCCC -3’ and BG553-F: 5′- ATTTGCGGCCGCTTTGTCACCTGGTTTGGCATC -3′, protective base GC and *Sal*I restriction site to the reverse primers BG380-R: 5′-GCGTCGACTTTGACAGGTGGAGAGGAACT-3′ and BG553-R: 5′-GCGTCGACGGTTTGCTTACAATGTGGTTCA-3′. The PCR was performed to amplify BG380 or BG553 cDNA in a 25 μl system from a 0.5 μl *B. glabrata* cDNA template. The reaction program was 95 °C for 5 min, followed by 35 cycles of 30 s at 95 °C, 30 s at 64 °C, and 3 min at 72 °C with a final extension of 10 min at 72 °C. In addition, the RNAi negative control was set as a 324 bp of enhanced green fluorescent protein (EGFP) gene (spanned position 21–444) named EGFP324. EGFP324 cDNA fragment was amplified from the pEGFP-NE1 plasmid (U55762.1) using the specific primers EGFP324-F: 5′- ATTTGCGGCCGCGGCAAGCTGACCCTGAAGT -3′ and EGFP324-R: 5′- GCGTCGACGTTGTAGTTGTACTCCAGCTTGT -3′, with a similar procedure as in amplification for BG380 and BG553. The amplified PCR products were purified separately and digested with restriction enzymes *Not*I and *Sal*I, then ligated into the L4440 plasmid vector. The recombinant BG380-L4440, BG553-L4440, and EGFP324-L4440 plasmids were transformed into the DH5α competent cells, respectively. The positive clones were screened by PCR and sequenced to confirm the correct sequences. The correct recombinant plasmids were transformed into RNase III defective HT115 cells to express BG380 dsRNA, BG553 dsRNA, and EGFP324 dsRNA, respectively. Meanwhile, the L4440 plasmid was transformed as the control. dsRNAs were induced at 37 °C for 4 h by adding 0.8 mM isopropyl-b-thiogalactopyranoside (IPTG) into the Luria–Bertani liquid medium containing ampicillin and tetracycline when the OD600 reached approximately 0.4–0.5. The sediment was concentrated by centrifugation at 10,000 g for 15 min, and the induced dsRNA was extracted from precipitation with Trizol reagent. The integrity and size distribution of dsRNA was evaluated using agarose gel electrophoresis, and RNA purity and concentration were determined using a spectrophotometer. The correct clone was screened out to amplify enough dsRNA.

### RNAi and SHAM treatment, investigation on snail growth and development

The RNAi treatment was performed on a clean bench at 26–28 °C with a 12 h light and 12 h dark photocycle. Snails were randomly selected and grouped into 3.0–5.0 mm, 7.5–8.5 mm, and 10.0–11.0 mm in diameter. The concentrated HT115 cells containing BG380-L4440, BG553-L4440, EGFP324-L4440, and L4440 plasmids were mixed into 1 mg dried snail feed and suspended in 1 ml RNAase-free water, respectively. The 200 μl mixture was fed to individual snails in each well of the six-well plate containing 8 ml RNAase-free water, with the final work concentration of 45.0 μg/μl and 112.5 μg/μl for HT115 cells. The solution changed daily, and snails were collected on the 2nd, 4th, and 6th days. In addition, the untreated control group was only fed with the quantified snail feed but without the cell mixture. At least six biological replicates of snails in each size treated with the same solution were collected for the RNA extraction and qPCR to evaluate the expression profile of *BgAOX* after the RNAi performance.

In addition, an AOX inhibitor, SHAM, dissolved in 0.0002% methanol at the final concentration of 0.2 mM, which was set as the optimal concentration by survival curve evaluation for snails treated with 0.1–1.6 mM SHAM, was mixed into the same snail feed as above and added to individual snails in each well of the six-well plate. The 0.0002% methanol and sterile water containing snail feed were separately set as treatment control for snails. The size of individual snails was calculated weekly, and egg production was checked daily. At least ten snails of the same size were investigated parallelly, and the treatment and investigation lasted for 4–5 weeks.

Based on the efficiency of RNAi and SHAM inhibition on snails of different sizes, the 3.0–5.0 mm sized snails were further selected and divided into seven groups with the difference in feeding solution, including the BG380 RNAi group (45.0 μg/μl HT115 containing BG380-L4440), BG553 RNAi group (45.0 μg/μl HT115 containing BG553-L4440), EGFP324 RNAi control group (45.0 μg/μl HT115 containing EGFP324-L4440), RNAi negative control group (45.0 μg/μl HT115 containing L4440), SHAM group (0.2 mM in 0.0002% methanol), SHAM control group (0.0002% methanol), and the blank control group (sterile water). The feeding solution changed daily, and the treatment lasted three weeks.

At least six snails of each group were collected parallelly on the 2nd, 4th, 6th, 8th, 11th, and 14th day to examine the efficiency of *BgAOX* mRNA knockdown using qPCR with the same procedure described above. The snail size was calculated for individual snails on the 1st and 21st days. Ten snails per group were investigated at each time point and analysed statistically. In addition, three snails in each group were taken out on the 14th and 21st day, respectively, then washed and dissected with the same procedure described before to perform traditional HE staining, observing mainly the head-foot, mantel, hepatic duct, ovotestis, yolk granules, and sperm. In addition, the head-foot, liver, and ovotestis of snails at the same time in each group were dissected and fixed in 2.5% glutaraldehyde at room temperature for 2 h. After osmic acid fixation, dehydration, embedding, and determining the location of head-foot, liver, and ovotestis by semithin sectioning and microscopic observation, the specimens were sectioned into 70 nm thick and stained with uranium acetate and lead citrate, observed under a transmission electron microscope (TEM) (HT7700-SS, Hitachi, Tokyo, Japan).

## Results

### The general morphological characters of *B. glabrata* snail during growth

**Fig. 1:**
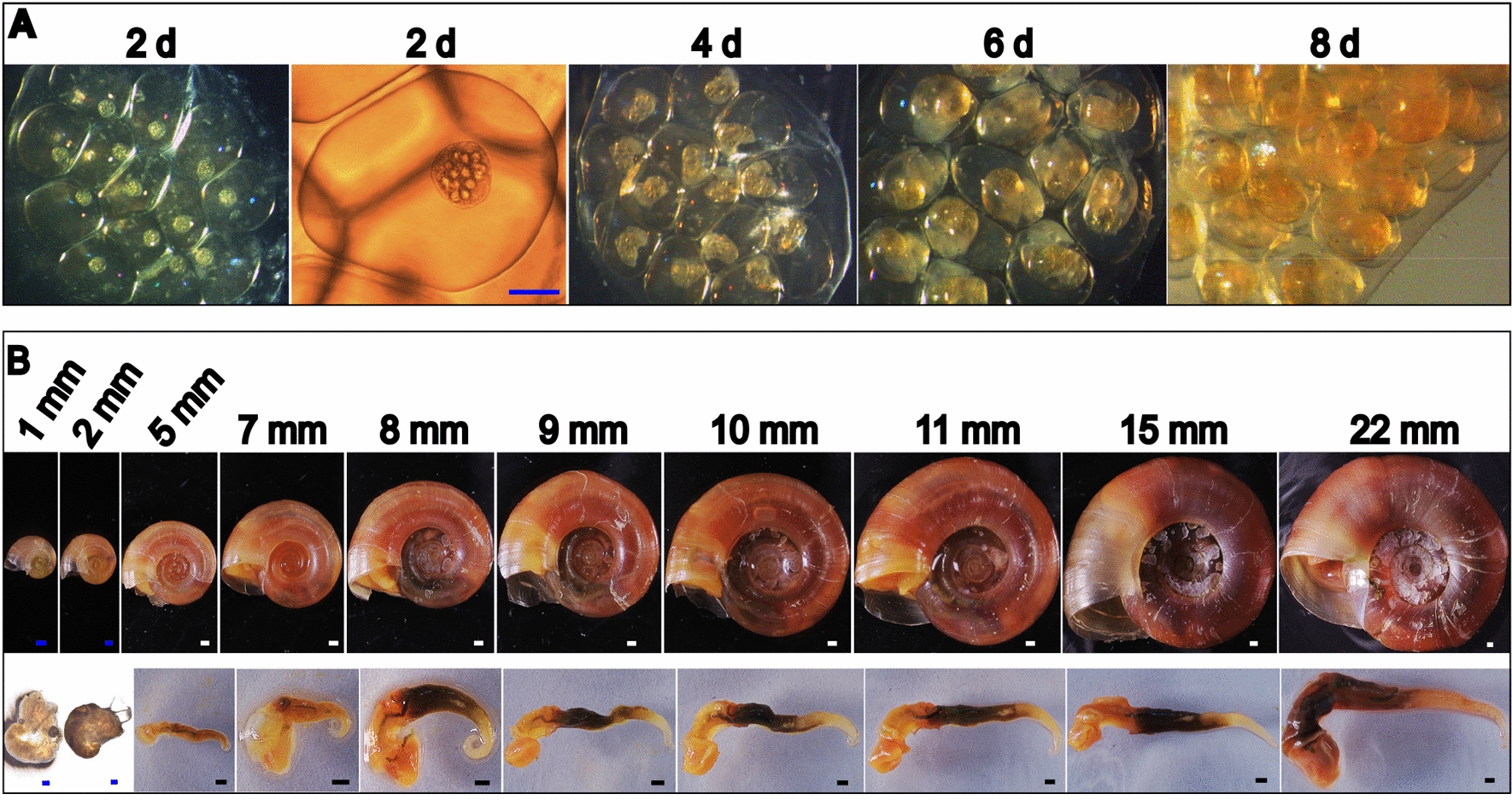
The appearance of snails in different sizes. **A** The egg clutches at 2 d, 4 d, 6 d, and 8 d after laying, which develops from cells to the larval stage of the snail. **B** Snails in size of shell diameter in 1.0 mm, 2.0 mm, 5.0 mm, 7.0 mm, 8.0 mm, 9.0 mm, 10.0 mm, 11.0 mm, 15.0 mm, and 22.0 mm, respectively. The upper row shows snails with shells arranged to be measured, and the lower row represents the soft body of the upper snail in the corresponding size. Scale bar in blue = 200 μm, scale bar in white = 500 μm, scale bar in black = 1 mm

Snail size was checked every month after hatching from egg clutches within two weeks (Fig. 1A). The growth rate varied much among *B. glabrata* snails of the same age. The size in diameter differed significantly at 2.0–4.0 mm, 3.0–7.0 mm, 3.0–15.0 mm, and 15.0–18.0 mm for snails of 1, 2, 3, and 4 months old, respectively. In addition, snails of the same size but different ages showed a similar appearance of vital organs. The juvenile snail of 1.0–2.0 mm (1-month-old) showed a yellowish-brown, thin, fragile shell. The soft body was gelatinous in translucent light-brown, and the head-foot, brown liver, and greyish-black heart with noticeable beating were visible under the microscope. However, ovotestis was not observed yet (Fig. 1B). During snail growth, shells became harder, and the colour gradually reddish (Fig. 1B). The soft body of the 2.0–3.0 mm (1-month-old) sized snail was gelatinous with brown colour. Ovotestis was still invisible (Fig. 1B). Snail of 5.0 mm (2 months old) was reddish-brown, with a yellow head-foot, reddish-brown liver, red heart, and greyish-green intestine observed with naked eyes. A small white ovotestis appeared at the end of the soft body (Fig. 1B). When snails grew to 7.0 mm (3 months old), the size of tissues, including the head-foot, liver, and ovotestis, significantly increased, and the end of the soft body curved to round (Fig. 1B). The 8.0–15.0 mm sized snails had a similar colour in yellow head-foot, brownish-black liver, and cream-yellow ovotestis, with the discoid or curved end (Fig. 1B). The 22.0 mm snails changed to slightly red, with distinct orange-red lines on the ovotestis (Fig. 1B).

### The dynamic and tissue-specific expression profile of the *BgAOX* gene

**Fig. 2:**
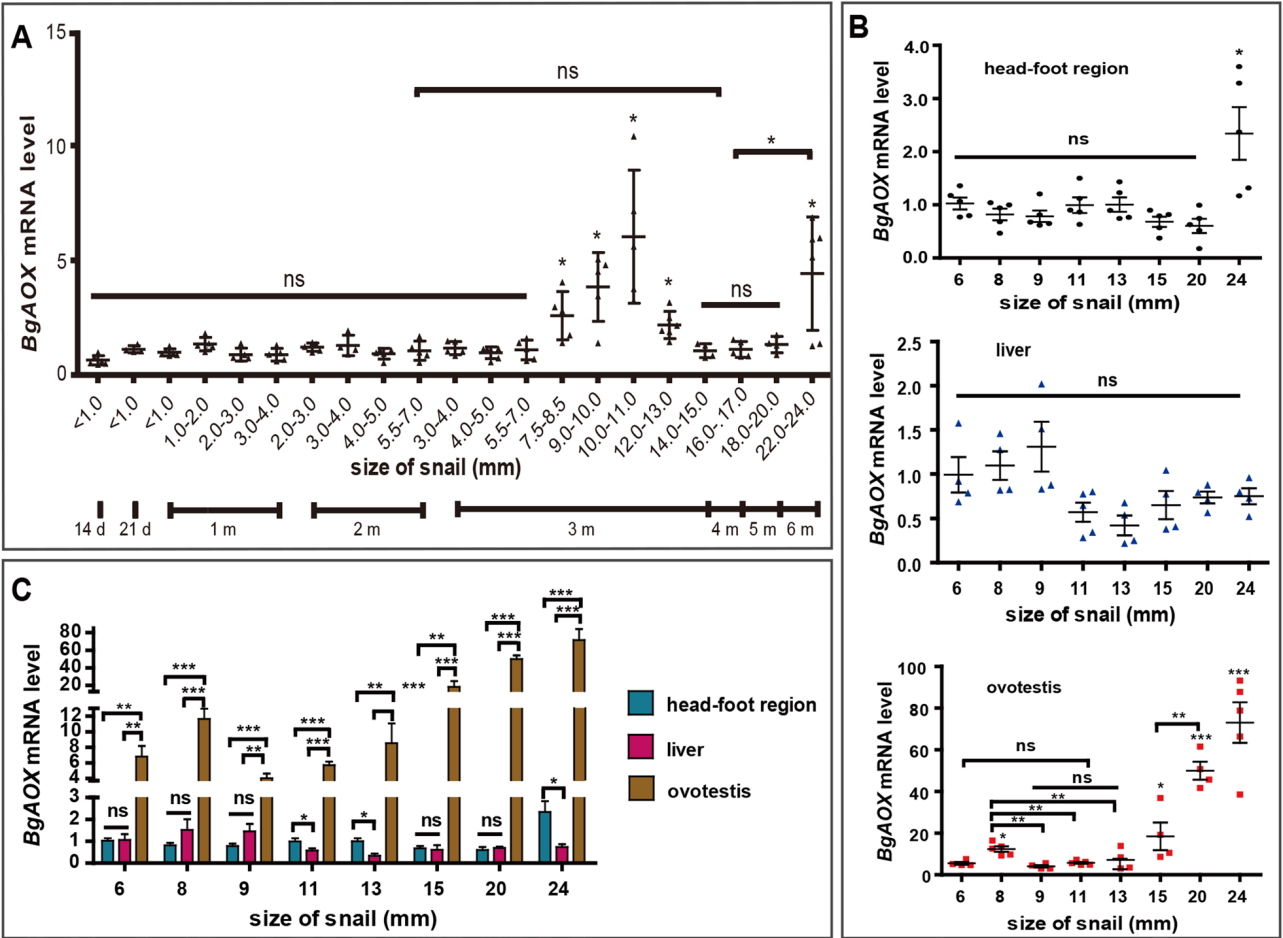
The dynamic and tissue-specific expression of the *BgAOX* gene in different-sized *B. glabrata* snails during growth. **A** The dynamic expression profile of *BgAOX* in the whole soft body of snails. The x-axis represents the size (mm in diameter) of snails, and the lower number means the age of corresponding-sized snails, from 14 days (d) to 6 months (m). The y-axis represents the fold change of *BgAOX* mRNA level in snails. *(*p* < 0.05) or ns (no significant difference) means comparison between the corresponding sized snail and 5.5–7.0 mm snail. Comparison between specific groups is marked with the beginning and ending points on the underlines. **B** The dynamic expression of *BgAOX* mRNA in specific tissues, including the head-foot, liver, and ovotestis of 6–24 mm snails. *(*p* < 0.05), **(*p* < 0.01), ***(*p* < 0.001) or ns means *BgAOX* mRNA amount comparison between the corresponding sized snail and 6.0 mm snail, comparison between specific groups is marked with the beginning and ending points on underlines. **C** The comparison of *BgAOX* mRNA amount among the head-foot, liver, and ovotestis in snails of the same size

The *BgAOX* gene relative expression profiles were firstly evaluated in the soft body of snails (Fig. 2A). No difference (ANOVA, *F*_(9, 40)_ = 2.034, *P* = 0.578) showed among juveniles from < 1.0 mm to 7.0 mm, with *BgAOX* in 5.5–7.0 mm snails setting as the average level. It increased significantly in 7.5–8.5 mm snails (t-test, *t*_(8)_ = 2.942, *P* = 0.019), kept increasing and reached the highest in 10.0–11.0 mm snails (t-test, *t*_(8)_ = 3.765, *P* = 0.006) (Fig. 2A). Then it decreased in 12.0–13.0 mm snails and then back to the average level in 14.0–20.0 mm snails (ANOVA, *F*_(3, 14)_ = 2.044, *P* = 0.681). However, it increased again in 22.0–24.0 mm snails, significantly higher than the level in juvenile snails (ANOVA, *F*_(10, 45)_ = 6.522, *P* = 0.013) (Fig. 2A).

Considering the *BgAOX* mRNA was significantly up-regulated at the time when snails grew to 7.5–8.5 mm, which is also the beginning time of oviposition for most snails, the dynamic expression profile of *BgAOX* in specific tissues, including the head-foot region, liver, and ovotestis was further investigated for 6.0–24.0 mm snails. *BgAOX* relative expression in the head-foot region showed no significant difference among 6.0–20.0 mm snails (ANOVA, *F*_(6, 28)_ = 2.334, *P* = 0.237) but significantly increased in 24.0 mm snails (t-test, *t*_(8)_ = 2.591, *P* = 0.032), with that in 6.0 mm snails setting as the control (Fig. 2B). No difference showed in the liver among all sized snails of 6.0–24.0 mm (ANOVA, *F*_(7, 25)_ = 2.021, *P* = 0.609) (Fig. 2B). The expression profile in the ovotestis differed from that in the head-foot region and liver. It increased significantly in 8.0 mm snails compared to that in 6.0 mm snails (t-test, *t*_(7)_ = 3.807, *P* = 0.011) (Fig. 2B), then decreased in 9.0–13.0 mm snails, back to the same level as that in 6.0 mm snails (ANOVA, *F*_(3, 13)_ = 1.012, *P* = 0.428) (Fig. 2B), and increased again and maintained the high level in 20.0–24.0 mm snails (ANOVA, *F*_(2, 10)_ = 24.030, *P* = 0.000) (Fig. 2B).

*BgAOX* expression profiles among different tissues in snails of the same size were compared with that in the head-foot of 6.0 mm snails. No significant difference showed between the head-foot region and the liver for snails of the same size, except for the 11.0 mm, 13.0 mm, and 24.0 mm snails in which the expression level in the head-foot was higher than the liver (ANOVA, *F*_(3, 26)_ = 3.554–7.478, *P* = 0.017–0.045) (Fig. 2C). It was evident that the ovotestis was the critical tissue to express *BgAOX*, with 5–23 times and 6–270 times higher than that in the head-foot and liver from the same-sized snails (ANOVA, *F*_(7, 58)_ = 10.003–40.964, *P* = 0.000–0.002), respectively (Fig. 2C).

### The oviposition behaviour and ovotestis development of snails with *BgAOX* gene expression changing

The egg production of snails in 6.0–24.0 mm was calculated in two weeks. Snails started to lay eggs when they grew to ~ 8.0 mm in diameter. With snail size increasing, the egg production of snails significantly increased (ANOVA, *F*_(6, 252)_ = 71.34, *P* = 0.000), from 0.07 eggs per snail per day for 8.0 mm snails to 61.07 eggs per snail per day for 24.00 mm snails (Additional file 1: Fig. S1A). Together with the *BgAOX* expression profile in ovotestis from 8.0 mm to 24 mm snails shown in Fig. 2B, the correlation analysis was performed between the average relative expression of *BgAOX* in ovotestis and the egg production of the snail. It showed that *BgAOX* relative expression level in ovotestis was positively correlated with egg production daily per snail of the same size, with a correlation coefficient (r) of 0.97 (Additional file 1: Fig. S1B).

**Fig. 3:**
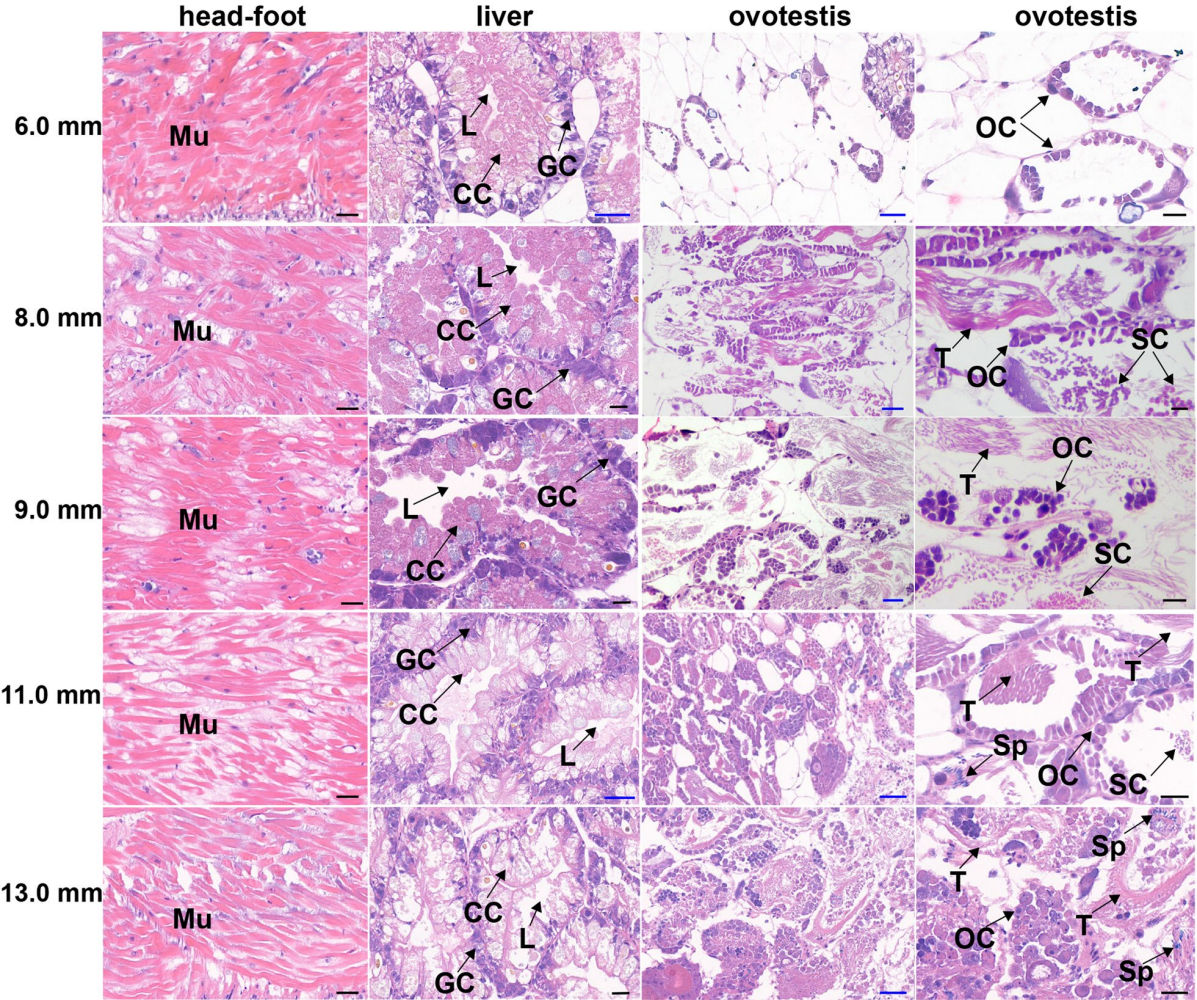
The morphology of different tissues, including the head-foot, liver, and ovotestis, in 6.0–13.0 mm snails with HE staining. Scale bar in black = 20 μm, scale bar in blue = 50 μm. *Mu* muscle, *L* lumen, *GC* granular cell, *CC* cylindrical cell, *OC* ovary cell, *T* testis, *SC* spermatic cord, *Sp* sperm. Two lines of ovotestis represent the same ovotestis with lower and higher magnification, respectively

The morphology of ovotestis in 6.0 mm, 8.0 mm, 9.0 mm, 11.0 mm, and 13.0 mm snails were observed with HE staining, with the head-foot region and liver from the same snail observed parallelly. In the ovotestis of the 6.0 mm snail, the limited number of ovarian cells in purple-red located closely to glandular tubes in the liver, but without testicle, spermatic cord, and sperm (Fig. 3). In 8.0 mm and 9.0 mm snails, testicles in red appeared, with numerous ovarian cells and spermatic cords in purple-red. Sperm in blue could be seen in 11.0 mm snails, with many ovarian cells and spermatic cords, which increased much more in 13.0 mm snails (Fig. 3). Muscle fibres in the head-foot were dense and regularly arranged in all-sized snails. The space among muscle bundles and the connective tissue gradually increased in 9.0–13.0 mm snails (Fig. 3). The liver of 6.0–13.0 mm snails contained regularly distributed glandular tubes, with irregular round dark blue granular cells distributed at the edge of the wall of glandular tubes, long cylindrical cells uniformly arranged at the basal layer of the glandular tubes (Fig. 3). The morphological changes in ovotestis during development from 6.0 mm to 13.0 mm snails were more significant than those in the head-foot and liver. The most change happened in ~ 8.0 mm snails.

### dsRNA-mediated knockdown of *BgAOX* in different-sized snails

The BgAOX-380/553-L4440 recombinant plasmid was constructed successfully and transformed into HT115 competent cells, with L4440 plasmid and EGFP324-L4440 plasmid as the control in parallel (Additional file 2: Fig. S2A). After induction by IPTG, the cells were collected, and the total RNA was extracted. The expected sized dsRNA band showed on the agarose gel, with total RNA of uninduced cells as the control (Additional file 2: Fig. S2B). After feeding the corresponding cells (45.0 μg/ml and 112.5 μg/ml) containing dsRNA, the knockdown efficiency of *BgAOX* mRNA was evaluated in 3.0–5.0 mm, 7.5–8.5 mm, and 10.0–11.0 mm snails, respectively. A significant decrease of *BgAOX* expression was observed in 90% (9/10) of 3.0–5.0 mm snails treated with BgAOX-380 dsRNA (45.0 μg/ml) at 2 days post-RNAi (ANOVA, *F*_(3, 21)_ = 8.979, *P* = 0.023), further to 0.23-fold transcript level for 100% of 3.0–5.0 mm snails at 6 d post-feeding (Additional file 3: Fig. S3A). For BgAOX-553 dsRNA treated snails, 60% (5/10) and 70–80% (7/10 in 45.0 μg/ml and 8/10 in 112.5 μg/ml) of 3.0–5.0 mm snails showed a significant decrease of *BgAOX* relative expression at 4 days and 6 days post-feeding, respectively (Additional file 3: Fig. S3B). Knockdown of *BgAOX* mRNA was limited for most 7.5–8.5 mm snails (ANOVA, *F*_(3, 8)_ = 3.364, *P* = 0.076), while 10–20% of snails showed efficient knockdown of *BgAOX* with BgAOX-380 dsRNAs at 2–6 days post-feeding (ANOVA, *F*_(3, 28)_ = 7.886, *P* = 0.041). Similarly, no significant decrease in *BgAOX* expression showed for 10.0–11.0 mm snails, except 40–60% (4/10 in BgAOX-380 dsRNA and 6/10 in BgAOX-553 dsRNA) of snails showed a significant decrease at 2 days post-feeding (Additional file 3: Fig. S3). These results suggested that BgAOX-380 dsRNA and BgAOX-553 dsRNA could efficiently decrease *BgAOX* mRNA in juvenile snails of 3.0–5.0 mm size but not very well in adult snails. In the following RNAi, 3.0–5.0 mm snails were used to observe the growth and morphological change for a longer time.

### Inhibition of BgAOX activity decreases the growth and oviposition of different-sized snails

**Fig. 4:**
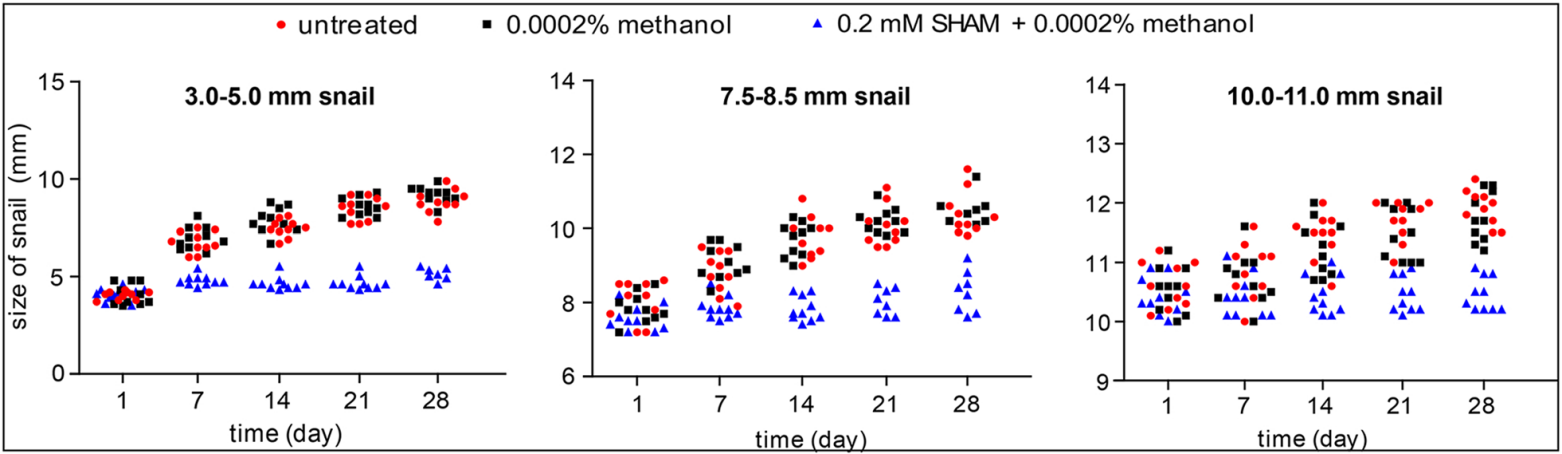
The effect of AOX inhibitor on snails’ growth in different sizes of 3.0–5.0 mm, 7.5–8.5 mm, and 10.0–11.0 mm

After SHAM treatment, the 3.0–5.0 mm snails showed less than 1% increase in size at 28 days, significantly smaller (ANOVA, *F*_(8, 132)_ = 20.337, *P* = 0.000) than snails in the controlled group (treated with 0.002% methanol and H_2_O) that both increased 50% on average size (Fig. 4). The 7.5–8.5 mm snails increased 12.5% in size at 28 days, significantly smaller (ANOVA, *F*_(8, 129)_ = 13.441, *P* = 0.000) than snails in the controlled group that increased 25% at 28 days (Fig. 4). The size of 10.0–11.0 mm snails increased by 10% after SHAM treatment at 28 d, significantly smaller (ANOVA, *F*_(8, 135)_ = 11.339, *P* = 0.000) than snails in the controlled that increased by 15% (Fig. 4). These results proved that inhibition of AOX activity could significantly decrease the growth of *B. glabrata*. This inhibition gradually weakened with the increase in snail size.

In addition, the egg production of snails was calculated during 35 days of treatment. Six of ten untreated snails of 7.5–8.5 mm size produced 187 eggs, and 40% (4/10) of snails in the methanol group had 99 eggs. In contrast, only 10% (1/10) of snails in the SHAM group made 11 eggs in total, with an 83.3% decrease in the number of producible snails and a 94.1% decrease in total egg amount (t-test, *t*_(20)_ = 9.348, *P* = 0.000) compared to untreated snails. Seven of ten untreated 10.0–11.0 mm snails produced 427 eggs, and 80% (8/10) in the methanol group laid 611 eggs. While 40% of snails (4/10) in the SHAM group laid 206 eggs, it significantly decreased by 51.8% in egg amount compared to the untreated group. It suggested that inhibiting AOX activity decreased snail fecundity, which was more significant in smaller snails.

### Knockdown of *BgAOX* mRNA and enzymatic inhibition of BgAOX disrupt the growth and ovotestis development of snails in morphology

**Fig. 5:**
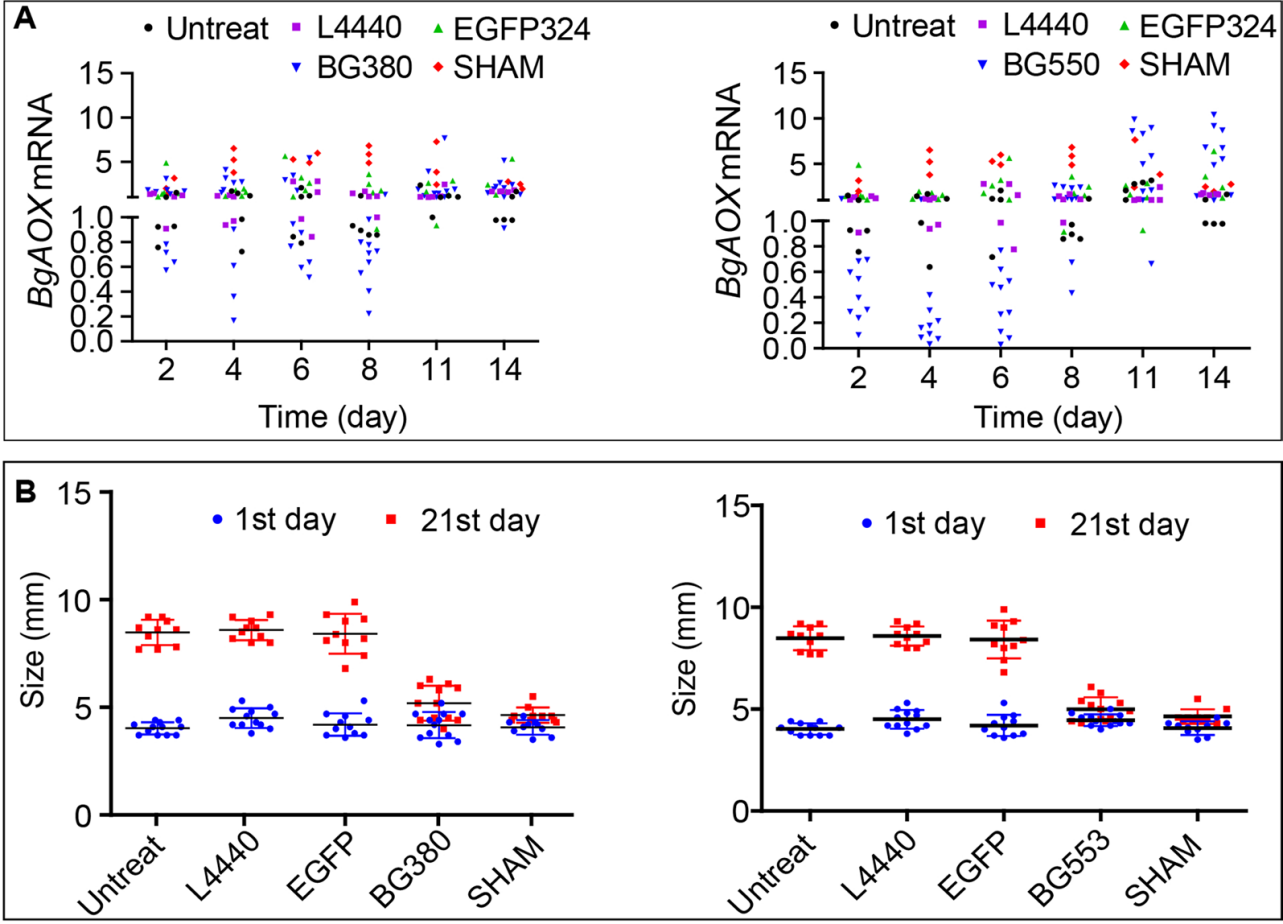
The RNAi efficiency and effect of AOX inhibition at mRNA level and enzyme activity on the growth of 3.0–5.0 mm snails. **A** The expression profile of *BgAOX* mRNA in snails after RNAi with Bg380 (left graph) and Bg553 (right graph). **B** The size change of snails after 21 days of RNAi (left graph with Bg 380 and right graph with Bg553) and AOX protein activity inhibition by SHAM

The 3.0–5.0 mm snails were treated with BgAOX-380/553 dsRNAs and 0.2 mM SHAM for three weeks, with the EGFP-324 group, L4440 plasmid group, and untreated group as the control. The *BgAOX* relative expression decreased significantly in 30% of snails at 2 days and 70% at 8 days after BgAOX-380 RNAi and back to the control level at 11 days and 14 days. Similarly, 90%, 90%, and 70% of snails in the BgAOX-553 RNAi group showed a significant decrease (ANOVA, *F*_(2, 33)_ = 15.20–38.43, *P* = 0.019–0.041) in *BgAOX* expression at 2, 4, and 6 days post-feeding, respectively, and increased back to the same level as the untreated group after 8 days post-feeding (Fig. 5A). It was noted that the *BgAOX* expression showed an immediate increase in snails after 2 days treated with 0.2 mM SHAM (Fig. 5A).

**Fig. 6:**
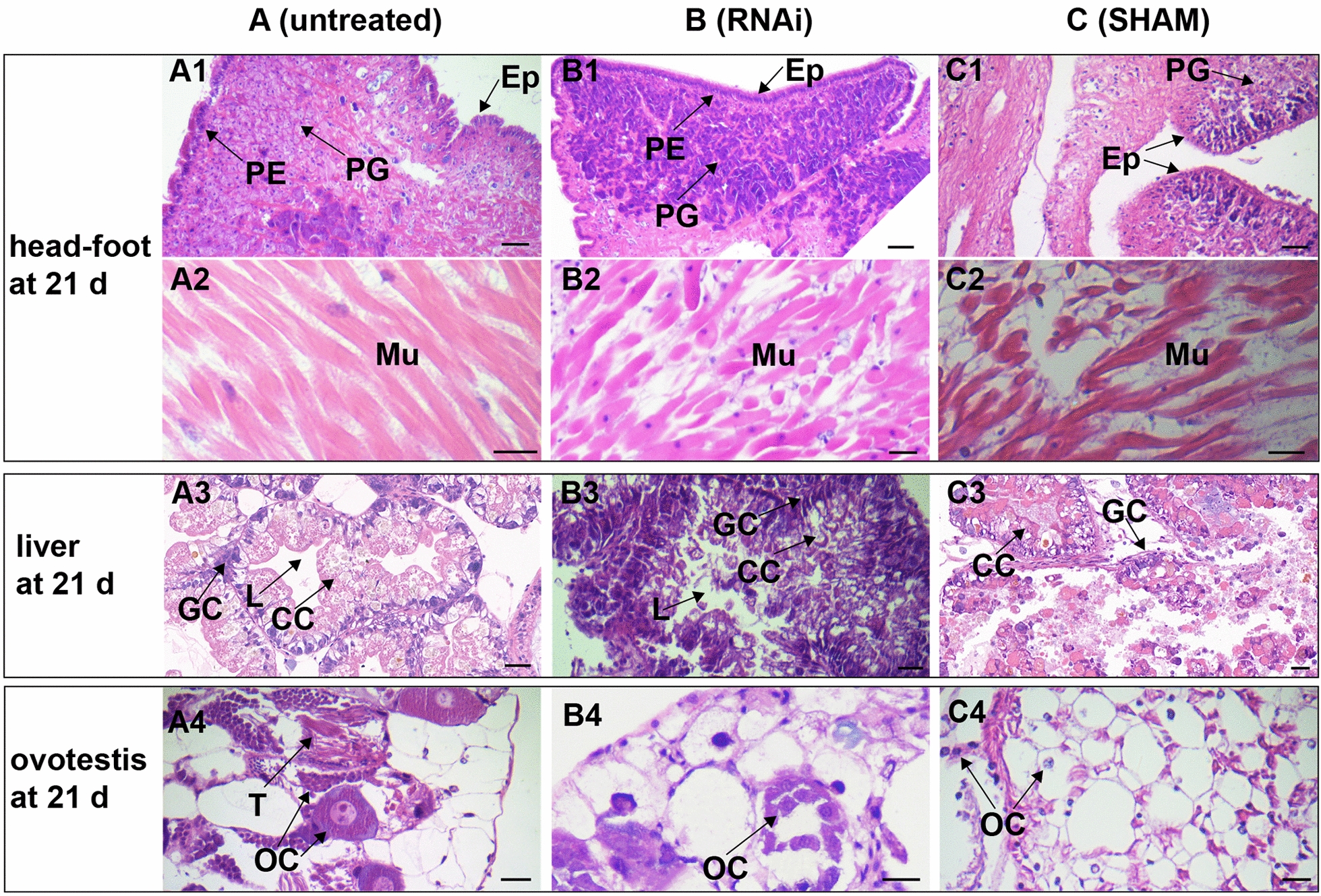
Microscopic images of different tissues in 3.0–5.0 mm snails after *BgAOX* mRNA knockdown and AOX activity inhibition. Scale bar in black = 20 μm. **A** Sections from untreated snails, including the head-foot region (A1, A2)**,** liver (A3), and ovotestis (A4) at 21 days. **B** Sections from RNAi-treated snails, including the head-foot region (B1, B2), liver (B3), and ovotestis (B4) at 21 days. **C** Sections from SHAM-treated snails, including the head-foot region (C1, C2), liver (C3), and ovotestis (C4) at 21 days. *PG* pedal gland, *Mu* muscle, *PE* pseudostratified epithelium, *Ep* epidermis, *GC* granular cell, *CC* cylindrical cell, *L* lumen, *OC* ovary cell, *T* testis

The growth of snails treated with dsRNAi and SHAM showed significant inhibition regarding the snail size. Snails in the untreated group, EGFP-324 group, and L4440 group showed no difference in size at 21 days post-treatment. Snail sizes in the Bg380 group, Bg553 group, and SHAM group all showed 50% smaller than the control groups (ANOVA, *F*_(4, 99)_ = 60.47–87.47, *P* = 0.000) (Fig. 5B). Snails in both the Bg380 group and Bg553 group were larger on average than in the SHAM groups but showed no significant difference (t-test, *t*_(2)_ = 2.106 or 2.825, *P* = 0.169 or 0.106).

In HE-stained untreated snails, plenty of pedal glands, intact epidermis with folds, regularly folded pseudostratified epithelium in the mantle collar, and tightly arranged muscle fibre with well-defined nuclei was observed in the head-foot region (Fig. 6A1, A2). After RNAi treatment, the pedal gland was still dense, and the pseudostratified epithelium in the mantle collar folded regularly. However, the epidermis became smooth, the folds disappeared, and the gap enlarged in muscle fibres (Fig. 6B1, B2). After SHAM treatment, the pedal glands ruptured and furtherly reduced, the folds on the epidermis disappeared, the pseudostratified epithelium in the mantle collar was irregularly arranged and turned blurred, and many vacuoles appeared and became larger among muscle fibres (Fig. 6C1, C2). In the liver of snails, granular cells contracted and gathered, vacuoles occurred in disordered cylindrical cells, the stellate lumen was blocked, and connective tissue was broken after RNAi (Fig. 6B3). After being treated with SHAM, granular cells in glandular tubes were vacuolated, the lumen was blocked, and connective tissue became disordered and even broken at 21 days post-treatment (Fig. 6C3). In the ovotestis of untreated snails, ovarian cells were abundant and arranged tightly (Fig. 6A4). After RNAi, both ovarian and sustentacular cells became disordered, intercellular space enlarged, and many ovarian cells spilt out of the tissue (Fig. 6B4). More sustentacular cells appeared after SHAM treatment, ovarian cells decreased, and some ovarian cells escaped through broken connective tissue (Fig. 6C4).

**Fig. 7:**
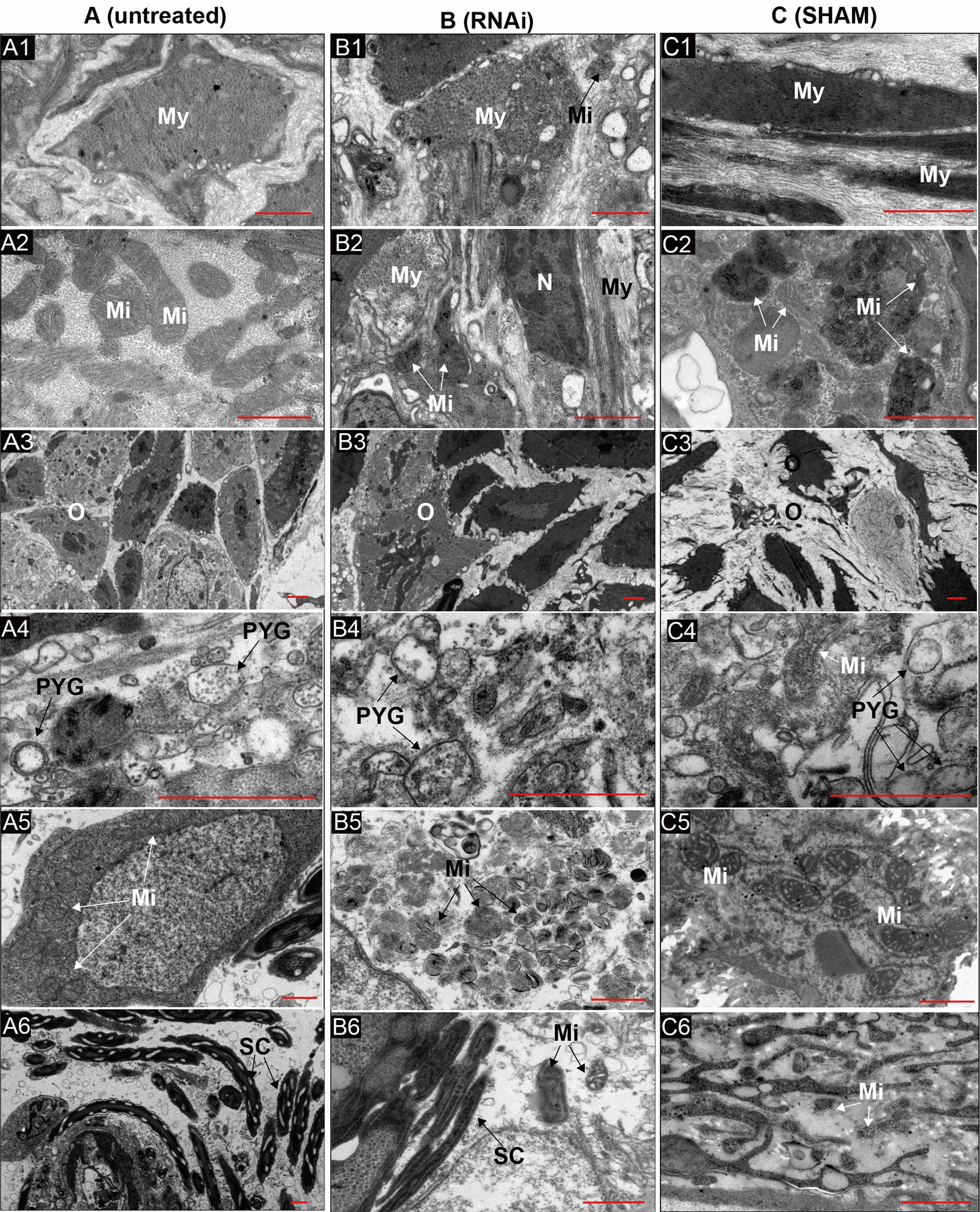
Ultrastructure images of the head-foot region and ovotestis of snails after treatments. Scale bar = 1 μm. **A** The muscle tissue (A1–A2) and ovotestis (A3–A6) of untreated snails. **B** The muscle tissue (B1–B2) and ovotestis (B3–B6) of snails after RNAi treatment. **C** The muscle tissue (C1–C2) and ovotestis (C3–C6) of snails after SHAM treatment. *My* myofilament, *Mi* mitochondria, *N* nucleus, *O* Ovary, *N* nucleus, *PYG* primary yolk granule, *SC* spermatic cord

Through TEM analysis, structural damages in the head-foot were furtherly investigated. Plenty of vacuoles appeared among broken muscle fibres, cell membrane ruptured, heterochromatin gathered and polarised, and cristae in mitochondrion became unclear and even broken, the endoplasmic reticulum fractured, and nuclei turned pyknotic with limited euchromatin after RNAi (Fig. 7B1, B2). After SHAM treatment, the muscle fibre became loose and disordered, plenty of vacuoles appeared in the cytoplasm, and chromatin gathered in high electron-dense, thickened mitochondrial cristae distributed unevenly and even disappeared (Fig. 7C1, C2).

In the ovotestis of untreated snails, ovarian cells were surrounded by connective tissue and arranged tightly and orderly, and the rough endoplasmic reticulum surrounded the primary yolk granulosa cells. The cell membrane was distinct, and chromatin was distributed evenly in the nucleus. Many mitochondria were detected and showed a high electron density matrix, with obvious cristae arranged regularly in the matrix (Fig. 7A3–A6). After RNAi and SHAM treatment, the space between ovarian cells enlarged and turned disordered, but more severely in SHAM treatment than in RNAi treatment (Fig. 7B3, C3). The yolk granules broke in ovotestis treated with RNAi (Fig. 7B4), while the endoplasmic reticulum dispersed around the yolk granules after SHAM treatment (Fig. 7C4). The cell membrane ruptured, vacuoles appeared in the cytoplasm, and heterochromatin was concentrated and gathered to the karyotheca in both treatments. The electron density in mitochondrial decreased, and cristae became fewer, disordered, and even disappeared after treatments (Fig. 7B5, C5). The boundary of the spermatic cord blurred after RNAi (Fig. 7B6). At 21 days post-SHAM treatment, intact cells were seldom observed, only left mitochondria without cristae and swollen endoplasmic reticulum without ribosome (Fig. 7C6).

## Discussion

The *BgAOX* expression profile varied with the growth and development of *B. glabrata* snail. The most important change happened from the juvenile to the adult stage. It increased significantly at the 7.5–8.5 mm stage since the birth of the snail, reached the highest level at the 10.0–11.0 mm snail, and then decreased after the 12.0–13.0 mm size. The ovotestis was identified as the primary tissue expressing *BgAOX* mRNA, 5–270 times higher than the head-foot region and liver from the same-sized snail. It was further proved that the *BgAOX* gene expression in ovotestis of 8.0 mm snails was highest among all ovotestis of 6.0–13.0 mm snails. It was noted that the 8.0 mm stage was the starting point of oviposition for most snails. These results made the 7.5–8.5 mm stage critical for *BgAOX* expression, especially in the reproductive system. With the high positive correlation (r = 0.975) between egg production and *BgAOX* expression profile in the same-sized snail ovotestis, the role of *BgAOX* in oviposition was further concerned*.* Morphologically, the most prominent development in ovotestis also happened in the 7.5–8.5 mm stage, with a large amount of regularly arranged ovarian and spermatic tissue, and spermatic cords, significantly different from the 5.5–7.0 mm stage, which had very few ovarian cells and without testis and spermatic cord. Meanwhile, little change showed in the head-foot and liver of snails from 6.0 to 24.0 mm for *BgAOX* gene expression and morphological development. It was concluded that the high expression of the *BgAOX* gene was involved in the growth and development of *B. glabrata* snail, especially the period from juvenile to adult, which is highly related to the ovotestis development and oviposition. This involvement may not only be associated with mitochondrial energy metabolism during growth, considering the *BgAOX* expression was not continuously increasing or decreasing.

Except for the high expression level in the 7.5–13.0 mm snails, *BgAOX* expression increased significantly in 24.0 mm snails. This increase mainly arose in the ovotestis and the head-foot in this stage, unrelated to the liver. The increase in the ovotestis of the 24.0 mm snails (6 months) was supposed to be helpful to the high egg production in this stage, which was highly positively correlated. The higher expression level of *BgAOX* in the head-foot of 24.0 mm snails may be induced by oxidative stress under specific tissue degeneration in snails getting older (≥ 6 months). AOX was confirmed to protect molluscs under stress [14, 34]. Further investigation is necessary to understand the role of AOX in aged snails facing survival stress.

The impact of AOX on the early stage of reproductive development and oviposition was further investigated by using *BgAOX* RNAi and AOX inhibitor, SHAM, which could inhibit AOX activity by blocking the binding of AOX to ubiquinone in the respiratory bypass of the mitochondrion. RNAi could significantly decrease *BgAOX* mRNA level and last for one week in 90–100% of 3.0–5.0 mm snails, although the *BgAOX* expression showed a reverse increase after a period of RNAi. This limited lasting period of non-invasive RNAi efficiency in juvenile *B. glabrata* snails also happened for other genes [35]. Meanwhile, the knockdown efficiency was limited and unsteady for most snails in 7.5–8.5 mm and 10.0–11.0 mm sizes. It was supposed to be related to the high expression profile of *BgAOX* mRNA in these two developmental stages, the response of compensational increase under RNAi stress, and more efficient physiological barriers in larger snails. In addition, the non-disruptive RNAi method was used in this study to introduce dsRNA into snails by feeding RNase III-deficient *E. coli* cells containing *AOX-*specific dsRNA, which may cause the unsteady RNAi knockdown efficiency but is optimal for the stress-related gene like AOX. This RNAi method was also proved to effectively silence the macrophage migration inhibitory factor gene in *O. hupensis* snails for four days [36].

Although *BgAOX* mRNA cannot be silenced well after one week of RNAi, the inhibition of snail growth and pathological damage to tissues took effect continuously. The growth rate of the 3.0–5.0 mm snail decreased significantly, 50% smaller in size after 21 days of RNAi than the untreated snail. The noticeable pathological changes were observed in the head-foot, liver, and ovotestis. These confirmed that the inhibition of AOX at the transcriptional level led to significant inhibition of the growth and development of snails. It was also noted that the pathological damage at 21 days post-RNAi was reduced compared with that at 14 days post-RNAi, which may be related to the weakened inhibition of *BgAOX* expression at the later stage of RNAi. It was reported that the cleft chest of steroid-dependent GeneSwitch *Drosophila* could be improved by high expression of AOX at transcriptional and protein levels [15]. In this study, the knockdown efficiency of *BgAOX* was weakened at the later stage of RNAi, and the expression of *BgAOX* returned to the average level and even increased, followed by the weakening of pathological damage, which suggested that the increased expression of *BgAOX* is helpful to repair the damage caused by RNAi. In addition, the expression of the *BgAOX* gene in the SHAM-treated snail was kept at a higher level. However, the morphological damage was significant and ongoing in the head-foot, liver, and ovotestis. The response under the SHAM-induced stress could explain the increase of *BgAOX* expression, and the tissue damage was led by the direct inhibition of AOX activity with SHAM.

After SHAM treatment, the growth rate of all 3.0–5.0 mm, 7.5–8.5 mm, and 10.0–11.0 mm snails were significantly decreased, and the oviposition capacity of 7.5–8.5 mm and 10.0–11.0 mm snails was also decreased. It confirmed the role of AOX activity in snail growth and the reproductive system. This phenomenon was also found in *Cryptosporidium parvum,* which is protozoa lacking mitochondrial classical respiratory chain, and in vitro growth of *C. parvum* was inhibited significantly after SHAM treatment [37]. Furthermore, this inhibition of growth and oviposition ability decreased gradually with the increase of the snail size. It implied that the younger the snail, the stronger the effect of AOX intervention on growth, development, and oviposition. The earlier intervention at the juvenile stage can delay the development of tissues, especially the ovotestis, which cannot be developed into matured and then unable to produce the egg, which could efficiently diminish the population quantity of this hermaphroditic snail.

## Conclusions

In this study, the impact of AOX on the growth and development of *B. glabrata* snail was confirmed, especially in reproductive development and egg production. Inhibiting AOX in transcript level and protein activity could lead to a systemic effect on snails’ growth, including oviposition, which may not only be related to mitochondrial energy metabolism but needs further investigation. The inhibition of AOX at the protein activity by SHAM could lead to more severe histological lesions and snail growth inhibition than at the transcriptional level by RNAi, although the AOX protein expression was not evaluated in this study. In previous studies, the AOX pathway activity in plants was mainly regulated at the post-translational level [38]. In addition, the AOX protein expression in *O. hupensis* was not coincident with the *AOX* mRNA expression, and post-translation regulation was also implied for the natural type of AOX in *O. hupensis* snail [14]. Then, the regulation mechanism of AOX activity deserves further investigation, which would help provide a potential target for developing an environmentally friendly snail control method.

## Supplementary Information

**Additional file 1: Figure S1.** The oviposition behaviour of snails with the *BgAOX* mRNA level changed. (A) The average number of egg production per snail per day in two weeks. (B) The correlation analysis between the egg production per snail per day and the average relative expression of *BgAOX* in ovotestis of snails of the same size.

**Additional file 2: Figure S2.** Identify target cDNA fragments from recombinant plasmids and target dsRNA from induced cells. (A) The PCR product of the target fragment from recombinant plasmid DNA. M, DL5000 DNA marker; Lane 1, the PCR product from L4440 plasmid; Lane 2, the EGFP324 fragment from plasmid EGFP324-L4440; Lane 3, the BG380 fragment from plasmid BG380-L4440; Lane 4, the BG553 fragment from plasmid BG553-L4440. (B) The extracted dsRNA product from HT115 cells contains the corresponding recombinant plasmid. M, DL5000 DNA marker; Lane 1 and 2, the product from uninduced and induced cells containing L4440 plasmids; Lane 3 and 4, the EGFP324 dsRNA (arrow) from uninduced and induced cells containing recombinant EGFP324-L4440 plasmid; Lane 5 and 6, the BG380 dsRNA (arrow) from uninduced and induced cells containing recombinant BG380-L4440 plasmid; Lane 7 and 8, the BG553 dsRNA (arrow) from uninduced and induced cells containing recombinant BG553-L4440 plasmid.

**Additional file 3: Figure S3.** The evaluation of the dsRNAi effect on different-sized snails. (A) BG380 dsRNAi treatments and other control groups. (B) BG553 dsRNAi treatments and other control groups. The treatments were distinguished in different symbols for each investigated time (day).

## Data Availability

Most data generated or analysed during this study are included in this published article and its additional information files. Any related data are available from the corresponding author upon reasonable request.

## Abbreviations

AOX: Alternative oxidase
WHO: World Health Organization
MDA: Mass drug administration
Cyt C: Cytochrome c
OhAOX: *Oncomelania hupensis* Alternative oxidase
BgAOX: *Biomphalaria glabrata* Alternative oxidase
RNAi: RNA interference
PR: Puerto Rico
qPCR: Quantitative real-time PCR
HE: Hematoxylin–eosin
dsRNA: Double-stranded RNA
SHAM: Salicylhydroxamic acid
EGFP: Enhanced green fluorescent protein
IPTG: Isopropyl-b-thiogalactopyranoside
