# Supplementary figures and images for: Inhibition of alternative oxidase disrupts the development and oviposition of Biomphalaria glabrata snails
Source: Parasit Vectors. 2023 Feb 17;16:73. doi: 10.1186/s13071-022-05642-8 (PMC9938623; doi:10.1186/s13071-022-05642-8)

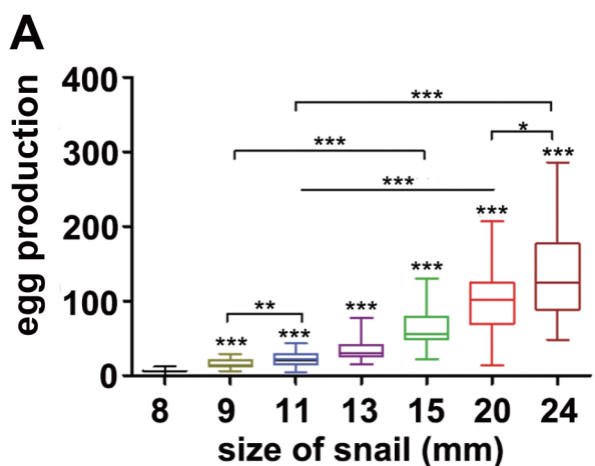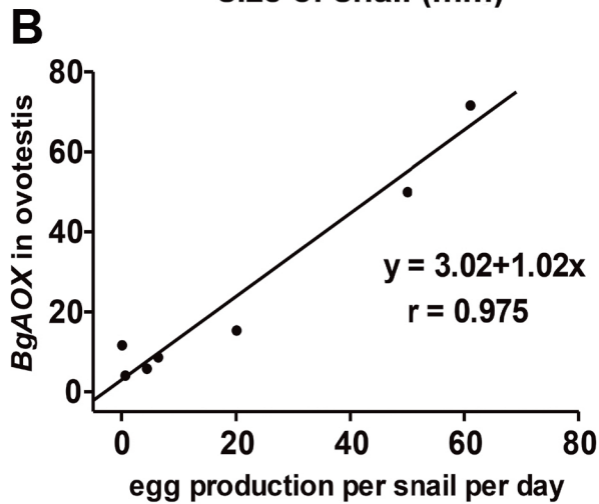

Supplement: Supplementary file 1 — Additional file 1: Figure S1. The oviposition behaviour of snails with the BgAOX mRNA level changed. (A) The average number of egg production per snail per day in two weeks. (B) The correlation analysis between the egg production per snail per day and the average relative expression of BgAOX in ovotestis of snails of the same size. [file 13071_2022_5642_MOESM1_ESM.pdf]

**A**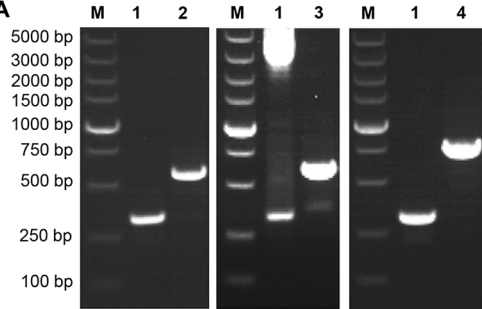**B**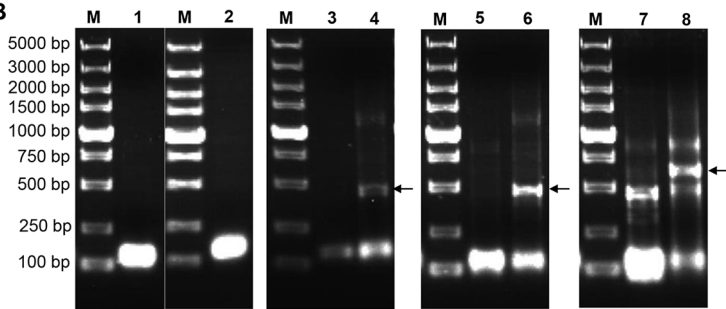

Supplement: Supplementary file 2 — Additional file 2: Figure S2. Identify target cDNA fragments from recombinant plasmids and target dsRNA from induced cells. (A) The PCR product of the target fragment from recombinant plasmid DNA. M, DL5000 DNA marker; Lane 1, the PCR product from L4440 plasmid; Lane 2, the EGFP324 fragment from plasmid EGFP324-L4440; Lane 3, the BG380 fragment from plasmid BG380-L4440; Lane 4, the BG553 fragment from plasmid BG553-L4440. (B) The extracted dsRNA product from HT115 cells contains the corresponding recombinant plasmid. M, DL5000 DNA marker; Lane 1 and 2, the product from uninduced and induced cells containing L4440 plasmids; Lane 3 and 4, the EGFP324 dsRNA (arrow) from uninduced and induced cells containing recombinant EGFP324-L4440 plasmid; Lane 5 and 6, the BG380 dsRNA (arrow) from uninduced and induced cells containing recombinant BG380-L4440 plasmid; Lane 7 and 8, the BG553 dsRNA (arrow) from uninduced and induced cells containing recombinant BG553-L4440 plasmid. [file 13071_2022_5642_MOESM2_ESM.pdf]

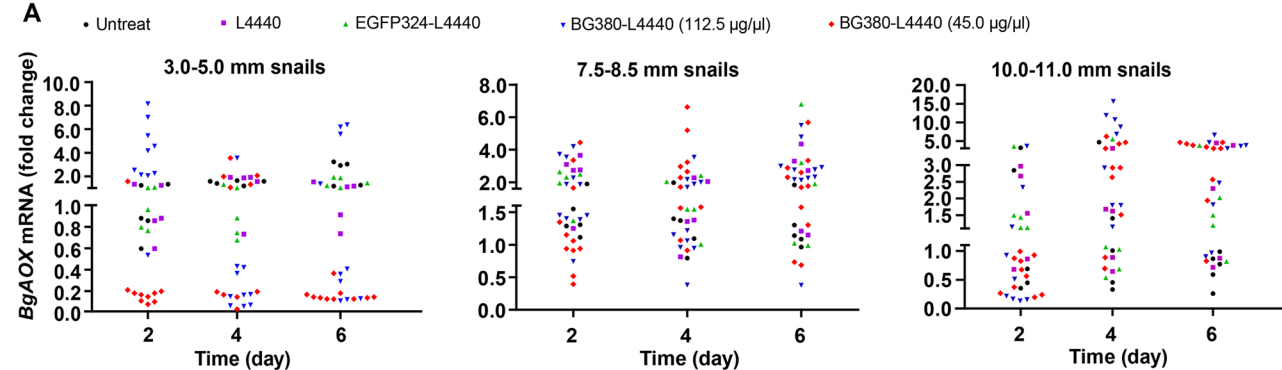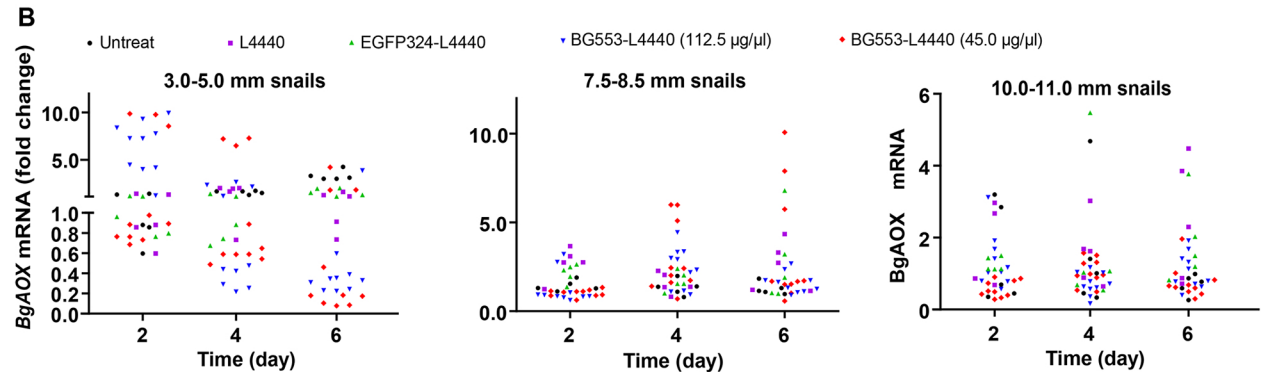

Supplement: Supplementary file 3 — Additional file 3: Figure S3. The evaluation of the dsRNAi effect on different-sized snails. (A) BG380 dsRNAi treatments and other control groups. (B) BG553 dsRNAi treatments and other control groups. The treatments were distinguished in different symbols for each investigated time (day). [file 13071_2022_5642_MOESM3_ESM.pdf]
